# Transcutaneous auricular vagus nerve stimulation after cesarean delivery: current evidence and trial design priorities

**DOI:** 10.3389/fmed.2026.1910274

**Published:** 2026-09-16

**Authors:** Qingjun Zeng, Haishan Cui, Shuang Guo, Chunmei Li, Yi Zhang

**Affiliations:** Department of Anesthesiology, Maternal and Child Health Hospital of Wanzhou District, Chongqing, China

**Keywords:** cesarean delivery, enhanced recovery after surgery, obstetric anesthesia, postoperative nausea and vomiting, postoperative pain, postpartum recovery, sham control, transcutaneous auricular vagus nerve stimulation

## Abstract

Post-cesarean recovery includes uterine contraction pain, incisional pain, nausea, sleep loss, breastfeeding concerns, and early mood symptoms. Transcutaneous auricular vagus nerve stimulation (taVNS) is a non-invasive neuromodulation method that has recently been tested after cesarean delivery. This review summarizes the current evidence and asks how future studies should be designed. A structured search of PubMed, Embase, Web of Science Core Collection, Cochrane CENTRAL, ClinicalTrials.gov, WHO International Clinical Trials Registry Platform, Chinese Clinical Trial Registry, China National Knowledge Infrastructure, and Wanfang was used to identify direct obstetric studies, selected perioperative studies from non-obstetric surgery, and mechanistic papers relevant to post-cesarean recovery. In total, 18 studies were retained. Only one adequately powered randomized trial directly tested taVNS after cesarean delivery. In that trial, 156 women undergoing elective cesarean delivery received active or sham taVNS once daily on the day of surgery and postoperative days 1 and 2. Active stimulation was associated with a lower incidence of moderate-to-severe uterine contraction pain on postoperative day 3. Early incisional pain, sleep, anxiety, and EPDS scores also favored active stimulation, but one-month anxiety and EPDS scores did not differ. Evidence for postoperative nausea and vomiting, ileus, lactation, and postpartum depression prevention remains indirect or trial-stage only. Current evidence supports further multicenter obstetric trials of taVNS, mainly for uterine contraction pain. It does not support routine clinical use. Future studies should use clear sham procedures, formal blinding assessment, objective biomarkers, lactation outcomes, and follow-up to at least 6 weeks postpartum.

## 1 Introduction

Cesarean delivery is a common major operation. After surgery, the mother has pain and nausea while she is also starting infant care and breastfeeding (1).

Analgesia after delivery must be chosen carefully. Many common analgesics can be used during breastfeeding when prescribed correctly. Opioids, however, can cause maternal sedation and may expose the neonate to small drug amounts through breast milk (2).

Nausea and vomiting around cesarean delivery are common and distressing. The risk is influenced by neuraxial opioids, hypotension after spinal anesthesia, uterotonic drugs, and other perioperative factors (3). In this review, postoperative nausea and vomiting (PONV) is treated as an important obstetric perioperative outcome, focusing on cesarean-specific perioperative nausea and vomiting.

Mood symptoms are another concern in the early postpartum period. A large global analysis estimated postpartum depression prevalence to be approximately 17%, with substantial variation between countries and study methods (4). Early depressive symptoms during hospital admission are clinically relevant, but they are not the same as a diagnosis of postpartum depression at 6 weeks or later.

For these reasons, Enhanced Recovery After Surgery pathways for cesarean delivery encourage multimodal recovery care and avoidance of unnecessary opioid exposure (5, 6). A safe non-pharmacological adjunct would be useful, but it must be tested directly in obstetric patients before clinical use.

taVNS stimulates the external ear at sites with vagal afferent innervation. The signal reaches the nucleus tractus solitarius and connects with autonomic, pain, and limbic networks (7–10). This biology provides a rationale for studying taVNS after cesarean delivery, but biological plausibility alone is not clinical evidence.

In 2025, Xiong et al. reported a randomized clinical trial of taVNS for uterine contraction pain after elective cesarean delivery (11). This trial is the direct obstetric anchor for the present review. We ask the following four questions: what direct obstetric evidence exists; what non-obstetric perioperative evidence is relevant but indirect; which mechanisms are plausible but unproven in postpartum patients; and how should future trials be designed?

## 2 Search approach and study selection

This is a review article, not a systematic review or clinical guideline. We used a structured search because the field is small and rapidly changing. The Scale for the Assessment of Narrative Review Articles (SANRA) was used as a reporting aid (12). A completed SANRA self-assessment is provided in Supplementary File S2.

We searched PubMed, Embase, Web of Science Core Collection, Cochrane CENTRAL, ClinicalTrials.gov, WHO International Clinical Trials Registry Platform, Chinese Clinical Trial Registry, China National Knowledge Infrastructure, and Wanfang from inception to mid-2026. No language or date restrictions were applied. Search terms included taVNS, auricular vagus stimulation, cesarean delivery, postpartum care, lactation, postoperative pain, PONV, ileus, Edinburgh Postnatal Depression Scale (EPDS), anxiety, oxytocin, and related terms. Full search strings are provided in Supplementary File S1.

Potential duplicate reports were identified by comparing titles, authors, publication year, DOI, and registry number before eligibility assessment. QZ screened titles and abstracts and then assessed potentially relevant full texts. YZ checked the final inclusion decisions. HC, SG, and CL checked the extracted information for assigned studies against the source reports. Differences were resolved by discussion with YZ. The review did not use fully independent duplicate screening.

Because this work was planned as a narrative review, a Preferred Reporting Items for Systematic reviews and Meta-Analyses (PRISMA)-style record log was not maintained. The exact number of records retrieved, removed as duplicates, and excluded at each stage therefore cannot be reported reliably. The final synthesis included 18 studies: one direct obstetric efficacy trial, one peripartum feasibility study, one obstetric trial protocol, eight indirect clinical studies from non-obstetric surgery, three preclinical mechanistic studies, and four methodological or mechanistic studies in healthy or psychiatric cohorts. Table 1 lists each study and its evidence category.

**Table 1:** Characteristics and evidence categories of the 18 studies included in this narrative synthesis.

Ref./First author (year)	Design	Population	Intervention/parameters	Sham or comparator	Primary outcome	Key finding
A. Direct obstetric efficacy evidence (*n* = 1)						
Xiong (2025) (11)	Double-blind sham-controlled RCT	156 women undergoing elective cesarean delivery under combined spinal-epidural anesthesia; approximately half were multipara	Left auricular concha; 20 Hz; 200 µs; 30 min once daily on surgery day and POD 1–2 after oxytocin	Same left-ear concha site with initial sensory ramp; device then off for remaining 30 min	Moderate-to-severe uterine contraction pain at POD 3	Pain incidence 5.1% vs. 28.2% (RR 0.18; 95% CI 0.07-0.50; *P* < 0.001). Early incisional pain, sleep, anxiety, and EPDS scores favored taVNS; 1-month EPDS/anxiety did not differ
B. Peripartum clinical feasibility evidence (*n* = 1)						
Deligiannidis (2022), DELOS-1 (13)	Multicenter open-label pilot trial	25 women within 9 months postpartum with peripartum MDD	Nesos wearable taVNS; 6-week treatment phase; parameters per original report	None (uncontrolled)	Change in HDRS-17	Open-label feasibility signal for diagnosed peripartum MDD; not evidence for prevention or routine obstetric use
C. Obstetric protocol-stage evidence (*n* = 1)						
Ji (2026) (14)	RCT protocol (no outcome data)	Cesarean-delivered women with antenatal depressive tendency	Perioperative taVNS; parameters per published protocol	Sham control per protocol	PPD incidence at 6 weeks	Registered protocol for PPD prevention after cesarean delivery; no outcome data available
D. Indirect clinical evidence from non-obstetric surgery (*n* = 8)						
Tsang (2011) (15)	RCT	Patients undergoing hysterectomy	Auricular TENS; parameters per original report	Inactive electrode placement	Acute postoperative incisional pain	Reduced acute somatic incisional pain; relevant to incisional pathway only
Patel (2023) (16)	Single-blind analyzer-masked RCT	Heterogeneous surgical procedures	Perioperative transauricular stimulation; parameters per original report	Sham placement without current	Postoperative pain and rescue opioid use	Reduction in postoperative pain and rescue opioid consumption
Zhang (2025) (17)	Participant- and assessor-blinded RCT	70 patients undergoing video-assisted thoracoscopic lobectomy	Perioperative + POD 1–2 stimulation; parameters per original report	Sham earlobe stimulation	Sufentanil consumption in first 24 h	No significant difference in opioid use, but significantly reduced resting and deep-breathing pain; indirect relevance only
Yin (2025) (18)	Randomized trial	96 patients undergoing perianal surgery	30-min pre-op + 30-min at 24 h post-op; parameters per original report	Sham stimulation	VAS scores at multiple postoperative timepoints	Lower VAS at early postoperative time points, lower rescue analgesia use, lower urinary retention
Wang (2026) (19)	RCT	94 patients undergoing glaucoma surgery under general anesthesia	Left auricular concha, twice daily 30 min from 2 days pre-op through POD 1; parameters per original report	Sham stimulation without current	Moderate-to-severe pain (NRS ≥4) within 24 h after surgery	Reduced moderate-to-severe pain and rescue acetaminophen use; improved early sleep. Ophthalmological surgery data are indirect for obstetrics
Ru (2023) (20)	Double-blind RCT	134 patients undergoing laparoscopic radical resection of colorectal cancer	Low-intensity pre-emptive taVNS; parameters per original report	Sham per original protocol	Time to first flatus/defecation; ileus incidence	Shortened time to first flatus and defecation; reduced postoperative ileus incidence
Li (2021) (21)	Observational cohort study	3,223 surgical patients: vagotomy vs. non-vagotomy	Not applicable (vagal anatomy contrast, not taVNS intervention)	Vagotomy vs. non-vagotomy comparator	Incidence of PONV	Vagotomy was associated with lower PONV; supports vagal pathway relevance but is not taVNS evidence
Shi (2026) (22)	Randomized sham-controlled trial	150 elderly patients undergoing major non-cardiac surgery	Cymba conchae from 5 min before induction through to the end of surgery; parameters per original report	Sham at earlobe	Postoperative delirium incidence in first 3 days	Lower delirium incidence in elderly non-cardiac surgery; indirect relevance only for obstetric patients
E. Preclinical mechanistic evidence (*n* = 3)						
Hong (2019) (23)	Murine surgical model	Mice with postoperative ileus and endotoxemia	Non-invasive auricular vagal stimulation	Sham stimulation	Gastrointestinal transit; inflammatory cytokines	Prevents postoperative ileus and attenuates endotoxemia via alpha-7 nAChR-mediated CAP
Go (2022) (24)	LPS-induced acute inflammation model in mice	Endotoxemic mice	taVNS at 15 Hz vs. 25 Hz; matched pulse parameters	15 Hz vs. 25 Hz parametric contrast	Serum TNF-alpha, IL-1beta; tissue injury markers	15 Hz produced greater suppression of pro-inflammatory mediators than 25 Hz; frequency-dependent CAP signal
Eliava (2016) (25)	Rat hypothalamic-spinal circuit characterization	Rat inflammatory pain model	Optogenetic and electrophysiological mapping	Not applicable	Parvocellular oxytocin neurons and spinal nociception	Parvocellular oxytocin neurons project to spinal dorsal horn and gate inflammatory pain; relevant to oxytocin-related pain hypothesis
F. Methodological and mechanistic evidence in healthy or psychiatric cohorts (*n* = 4)						
Austelle (2025) iWAVE (26)	Open-label pilot trial	10 inpatient psychiatric patients with comorbid depression and anxiety	Accelerated taVNS dosing schedule; parameters per original report	None (open-label feasibility)	PHQ-9, BDI, GAD-7, BAI	Feasibility and symptom signal for accelerated protocols; not peripartum prevention evidence
Zhu (2022) (27)	Mechanistic clinical study	Healthy adult volunteers	Standard taVNS clinical parameters	Earlobe sham	Salivary oxytocin; eye-gaze to facial features	Increased salivary oxytocin and altered social attentional bias; supports possible oxytocin-related mechanism only
Cheng (2025) (28)	Randomized crossover trial	Healthy adults under sleep-deprivation paradigm	Earlobe stimulation vs. active cymba; typical clinical intensities	Within-subject crossover	Cortical activity, alertness, HRV, EEG power spectra	Earlobe stimulation is not physiologically inert; important for trials using earlobe sham controls
Schiweck (2025) (29)	Single-blind crossover RCT	110 participants (51 controls, 59 MDD)	Right cymba concha vs. earlobe sham; acute stress paradigm	Earlobe sham; within-subject crossover	Cardiac, emotional, inflammatory reactivity to stress; baseline RMSSD stratification	Baseline RMSSD stratifies taVNS responsiveness; supports HRV as candidate biomarker

The studies are separated by evidentiary role. Only the randomized trial by Xiong et al. provides direct evidence of efficacy after cesarean delivery.

BAI, Beck Anxiety Inventory; BDI, Beck Depression Inventory; CAP, cholinergic anti-inflammatory pathway; EPDS, Edinburgh Postnatal Depression Scale; GAD-7, Generalized Anxiety Disorder-7; HDRS, Hamilton Depression Rating Scale; HRV, heart rate variability; ISI, Insomnia Severity Index; MDD, major depressive disorder; NRS, Numeric Rating Scale; PCIA, patient-controlled intravenous analgesia; PHQ-9, Patient Health Questionnaire-9; POD, postoperative day; PONV, postoperative nausea and vomiting; PPD, postpartum depression; RCT, randomized controlled trial; RMSSD, root mean square of successive differences; RR, relative risk; taVNS, transcutaneous auricular vagus nerve stimulation; TENS, transcutaneous electrical nerve stimulation; TNF, tumor necrosis factor; VAS, visual analog scale.

The evidence categories are descriptive. Non-obstetric, psychiatric, healthy-volunteer, protocol-stage, and animal studies are not treated as direct evidence for routine post-cesarean use.

We included studies in pregnant or postpartum populations when available. We also included selected non-obstetric perioperative studies when their outcomes were relevant to post-cesarean recovery, such as postoperative pain, ileus, nausea, or delirium. Preclinical and mechanistic papers were included only when they helped interpret biological plausibility. We did not pool results because populations, stimulation protocols, and outcomes were too different for a meaningful meta-analysis.

## 3 Direct obstetric and peripartum evidence

Table 1 accounts for all 18 included studies: the direct obstetric randomized efficacy trial by Xiong et al. (11), the DELOS-1 open-label feasibility study in peripartum major depressive disorder (13), one protocol-stage report of postpartum depression prevention after cesarean delivery (14), eight indirect clinical studies from non-obstetric surgery, three preclinical mechanistic studies, and four methodological or mechanistic studies in healthy or psychiatric cohorts. Only the trial by Xiong et al. provides efficacy data for post-cesarean recovery.

Postpartum uterine contraction pain is not limited to cesarean delivery. It has also been described after vaginal birth, and its severity may be influenced by obstetric factors such as parity and breastfeeding-related uterine contraction (30). This background supports the clinical relevance of afterpains, but it should not be used as a cesarean-specific comparator.

Xiong et al. enrolled 156 women scheduled for elective cesarean delivery under combined spinal-epidural anesthesia (11). Participants were aged 18 years or older, had singleton pregnancies at 37–42 weeks of gestation, and had American Society of Anesthesiologists physical status II or III. The trial included women who had delivered previously; approximately half of each group were multiparous.

Active stimulation was delivered to the concha of the left ear using a tVNS501 device (Changzhou Ruishen'an Medical Devices Co Ltd, Changzhou, China). The active protocol used 20 Hz, 200 µs, and 30 min per session once daily on the day of surgery and postoperative days 1 and 2. The stimulation current was increased for 30 s until tingling was reported, then decreased to a level slightly below that threshold. The sham protocol used the same electrode site and the same initial sensory ramp, but the device was then turned off for the remaining 30 min (11).

The primary outcome was moderate-to-severe uterine contraction pain on postoperative day 3. Active taVNS was associated with a lower incidence than the sham treatment [5.1% (4 of 78) versus 28.2% (22 of 78); RR 0.18; 95% CI 0.07–0.50; *P* < 0.001] (11). Secondary early outcomes also favored active stimulation, including T7 incisional pain, T7 EPDS score, T7 Pregnancy-Related Anxiety Questionnaire-Revised 2 score, T6 Leeds Sleep Evaluation Questionnaire sleep score, and T7 Obstetric Quality-of-Recovery Score recovery score. Patient-controlled intravenous analgesia (PCIA) use at 24 and 48 h was also lower in the active group. These secondary outcomes should be interpreted as early signals, not as proof of longer-term postpartum benefit.

The trial by Xiong et al. did not show a significant difference in breastfeeding status during the index admission. However, it was not a detailed lactation study. It did not measure milk transfer, infant intake, maternal prolactin, or milk cortisol. EPDS and anxiety scores did not differ between groups at the 1-month follow-up. Therefore, the trial should not be cited as evidence that taVNS prevents postpartum depression or improves lactation.

## 4 Evidence from non-obstetric perioperative studies

Several perioperative taVNS or auricular stimulation studies in non-obstetric surgery have reported effects on pain or recovery outcomes. These studies are useful for hypothesis generation. They should not be treated as direct evidence for cesarean patients.

For pain, randomized trials in thoracoscopic lobectomy, perianal surgery, glaucoma surgery, hysterectomy, and mixed surgical cohorts have reported reductions in some postoperative pain measures or rescue analgesic use (15–19). These findings support the hypothesis that auricular stimulation may influence postoperative pain pathways. However, post-cesarean uterine contraction pain is a specific visceral pain problem, and the relevance of non-obstetric somatic pain trials is limited.

For gastrointestinal recovery, Ru et al. reported shorter time to bowel recovery and less postoperative ileus after laparoscopic colorectal surgery (20). Animal studies support a possible cholinergic anti-inflammatory mechanism (23, 24). No trial has yet tested this after cesarean delivery.

For PONV, the evidence is anatomical and indirect. An observational cohort comparing vagotomy and non-vagotomy surgery supports a role for the gut–vagus–brain pathway in emesis (21). However, this does not prove that taVNS prevents PONV after cesarean delivery. Future obstetric trials should measure PONV as a prespecified outcome if this claim is to be tested.

For postoperative cognitive recovery, one randomized trial in elderly major non-cardiac surgery reported less postoperative delirium with auricular vagus stimulation (22). This is not directly applicable to postpartum women, who are younger and have different physiology. It does, however, support measuring sleep and autonomic outcomes in future studies (31).

## 5 Peripartum mood and lactation outcomes

The trial by Xiong et al. reported lower EPDS scores during admission, but not at 1 month (11). A postoperative day 3 EPDS score is an early symptom measure. It should not be described as postpartum depression prevention. A conventional postpartum depression outcome needs follow-up to at least 6 weeks and preferably longer.

The DELOS-1 trial studied a wearable taVNS device in women with major depressive disorder of peripartum onset (13). It was an open-label proof-of-concept trial and supports feasibility, not efficacy for prevention. The iWAVE pilot trial supports feasibility of accelerated taVNS in psychiatric inpatients, but is not evidence of peripartum depression prevention (26). A randomized trial protocol has been published to test preventive taVNS in cesarean-delivered women with antenatal depressive tendencies (14). Outcome data from this trial are not yet available.

Postpartum mental healthcare is broader than neuromodulation. Psychological care, physical activity, social support, and technology-assisted approaches are also being studied. A recent Research Square preprint reviewed this wider range of interventions (32). Because the report has not undergone peer review, it is cited only as contextual literature and not as evidence of taVNS efficacy.

Lactation is an important safety issue and the current evidence is weak. The trial by Xiong et al. reported breastfeeding status, but did not provide objective milk-transfer or infant-feeding physiology data (11). Future trials should include breastfeeding continuation, objective milk transfer when feasible, infant weight change, and maternal or milk biomarkers if available.

## 6 Mechanistic interpretation

Auricular vagus stimulation can activate central vagal projections (9). These projections connect with autonomic, inflammatory, pain, and stress-related pathways (7–10). These mechanisms are reasons to study taVNS, not proof that it improves post-cesarean recovery.

One proposed explanation for reduced uterine contraction pain involves central oxytocin pathways. Vagal stimulation may engage hypothalamic pathways, and animal research shows that parvocellular oxytocin neurons can modulate spinal nociception (25, 27). This mechanism remains a hypothesis in postpartum patients.

Another proposed explanation involves stress and autonomic balance. Pain, anxiety, sleep loss, and breastfeeding difficulty may interact through sympathetic and hypothalamic-pituitary-adrenal activity. taVNS may alter these pathways, but direct postpartum biomarker data are lacking. Future trials should measure heart rate variability, salivary cortisol, and relevant clinical outcomes if these mechanisms are claimed.

## 7 Sham control and risk of bias

Sham design is a major problem in taVNS trials. Some studies have used earlobe stimulation as a sham because the earlobe has less vagal innervation than the cymba conchae. This does not mean it is physiologically inert. Cheng et al. showed that earlobe stimulation can change alertness, cortical activity, and autonomic measures (28). This finding is important for taVNS trials that use earlobe stimulation as a control.

The trial by Xiong et al. did not use an earlobe sham. They used the same left-ear concha electrode site and an initial sensory ramp, followed by device-off stimulation for the rest of the session (11). This design avoids a simple earlobe-sham problem, but blinding was still not formally tested. Participants may have noticed differences in ongoing sensation, and outcome assessors did not report Bang’s Blinding Index or a similar measure (33).

The wider taVNS research field also has problems with small samples, variable parameters, and inconsistent biomarkers (34, 35). Minimum reporting standards for taVNS research should be followed, including stimulation site, waveform, pulse width, current, intensity titration, electrode design, device model, and sham protocol (36).

## 8 Certainty of evidence

Table 2 gives a Grading of Recommendations Assessment, Development, and Evaluation (GRADE)-informed appraisal of the main obstetric trial. The cumulative certainty for taVNS to reduce moderate-to-severe post-cesarean uterine contraction pain is low.

**Table 2:** Domain-by-domain GRADE-informed certainty appraisal of the main obstetric trial.

GRADE domain	Judgement	Rationale
Risk of bias	Serious (−1 level)	Randomization and allocation concealment were reported, and participants, study staff, and outcome personnel were described as blinded. The sham used the same left-ear concha site with an initial sensory ramp followed by device-off stimulation. Blinding success was not formally measured, and the primary outcome was participant-reported pain. Residual expectation or performance bias therefore cannot be excluded
Inconsistency	Cannot be assessed (no downgrade)	Only one adequately powered obstetric efficacy trial is available for post-cesarean uterine contraction pain (11). Between-study inconsistency cannot be estimated
Indirectness	Serious (−1 level)	The trial was conducted at one center in selected patients undergoing elective cesarean delivery with a uniform combined spinal-epidural anesthesia and analgesia pathway. Applicability to emergency cesarean delivery, other anesthetic pathways, different populations, and other health systems remains uncertain
Imprecision	Not serious for the primary outcome	The primary estimate was large and excluded the null (RR 0.18; 95% CI 0.07–0.50). No downgrade was applied for the primary outcome. Estimates for secondary outcomes, uncommon adverse events, lactation, and longer-term mood outcomes are less precise and should not be interpreted as confirmed benefits
Publication bias	Cannot be assessed (no downgrade)	Publication bias cannot be evaluated from one obstetric trial. The wider taVNS literature contains small studies and variable protocols, but this field-level concern cannot be quantified for the obstetric outcome

Narrative assessment of Xiong et al. (2025) (11) for moderate-to-severe uterine contraction pain on postoperative day 3.

Randomized evidence was considered high at the starting point. Certainty was downgraded one level for risk of bias and one level for indirectness, giving an overall low rating. Inconsistency and publication bias could not be assessed from one trial. The primary estimate was not downgraded for imprecision because its confidence interval excluded the null; secondary, safety, lactation, and longer-term outcomes remain imprecise.

The low rating means that the current evidence is not strong enough for routine clinical use. The main reasons are the single-center design; short follow-up; lack of formal blinding assessment; uncertain reproducibility; and limited evidence for safety, lactation, and longer-term mood outcomes.

Table 3 summarizes a research framework for future obstetric taVNS studies. It is not a treatment protocol. The table separates direct single-trial evidence from secondary, indirect, preclinical, and trial-stage evidence.

**Table 3:** Research questions and candidate parameter ranges for future obstetric taVNS studies.

Research area	Candidate question	Parameter range to test	Reason to study	Current supporting evidence	Evidence status	Key design caution
Preoperative anxiety and autonomic state	Can one preoperative taVNS session reduce anxiety or improve autonomic stability before cesarean delivery?	Auricular concha or cymba conchae; low-frequency 1–10 Hz; 200–250 μs; low sensory or sub-sensory stimulation; 20 min	Anxiety and sympathetic tone may affect pain, nausea, sleep, and breastfeeding after delivery	General taVNS physiology and reporting recommendations (34, 36); no obstetric efficacy trial	Hypothesis only	Do not claim clinical benefit without a powered obstetric trial
Uterine contraction pain	Can taVNS reduce moderate-to-severe afterpains after cesarean delivery?	Protocol close to Xiong et al.: left auricular concha; 20 Hz; 200 μs; 30 min once daily during surgery day/POD 1–2; sham method prespecified	This is the only outcome with direct randomized obstetric evidence	Xiong et al. (11), with mechanistic support from non-obstetric or animal studies (25, 27)	Direct single-trial signal	Use multicenter design, formal blinding assessment, and standardized analgesia
Incisional pain and opioid use	Does taVNS reduce somatic incisional pain or opioid use after cesarean delivery?	Probably similar to uterine-pain protocol, but dose-finding is needed	A secondary signal exists in the obstetric trial and several surgical pain trials	Secondary outcomes in Xiong et al. (11); non-obstetric trials (15–19)	Secondary or indirect signal	Separate visceral afterpain from incisional pain in outcomes and analysis
Bowel recovery and postoperative ileus	Does lower-frequency taVNS improve bowel recovery after cesarean delivery?	Auricular concha/cymba; ≤15 Hz; 200–250 μs; timing and intensity need dose-finding	Vagal anti-inflammatory mechanisms may influence postoperative ileus	Preclinical studies (23, 24) and colorectal surgery trial (20)	Indirect/preclinical support	Obstetric bowel recovery endpoints must be prespecified; avoid extrapolating from colorectal surgery
PONV	Can taVNS reduce PONV after cesarean delivery when added to standard prophylaxis?	Not established; 15–20 Hz could be explored only in trial settings	The vagal afferent system is involved in emesis pathways, but obstetric taVNS data are absent	Cesarean PONV background (3); observational vagotomy data (21)	Indirect evidence only	Do not describe taVNS as anti-emetic therapy unless obstetric trial data support it
Postpartum mood and sleep	Can taVNS improve longer-term postpartum depressive symptoms or sleep after cesarean delivery?	Home-use protocol not determined; parameters should follow trial protocols	Early mood scores improved in one trial, but 1-month scores did not differ and prevention of postpartum depression is unproven	Xiong secondary endpoint (11); DELOS-1 feasibility trial (13); iWAVE feasibility (26); protocol evidence (14)	Trial-stage only	Use EPDS or diagnostic outcomes at 6 weeks or later; do not equate POD 3 EPDS with PPD prevention

This table is a research planning aid only. It is not a clinical protocol or a recommendation for routine care.

EPDS, Edinburgh Postnatal Depression Scale; POD, postoperative day; PONV, postoperative nausea and vomiting; PPD, postpartum depression; taVNS, transcutaneous auricular vagus nerve stimulation.

Evidence status wording is intentionally conservative. Candidate parameters should be tested only in approved research protocols with device reporting consistent with published taVNS reporting standards (36).

## 9 Trial design priorities

Future studies should first confirm the effect on uterine contraction pain after cesarean delivery. Multicenter recruitment is needed, with clear analgesic co-intervention protocols and prespecified primary outcomes.

Sham control should be tested and reported carefully. Options include ramp-down stimulation at the active site or other validated approaches that preserve blinding without adding physiological treatment. Trials should measure blinding with a validated tool.

Trials should measure outcomes that matter to mothers and clinicians, such as uterine contraction pain, incisional pain, opioid use, PONV, bowel recovery, breastfeeding, sleep, anxiety, EPDS at discharge, and postpartum depression at 6 weeks or later. Safety outcomes should include uterine tone or fundal recovery, bleeding or lochia, bradycardia, skin events, headache, and infant outcomes.

Biomarkers may help show whether the intervention engages the intended pathways. Candidate measures include heart rate variability, salivary cortisol and, in selected studies, oxytocin-related measures. Baseline autonomic state may also help identify patients more likely to respond (29).

## 10 Conclusions

At present, taVNS after cesarean delivery is a research question, not a clinical standard. One randomized trial suggests benefit for early uterine contraction pain, but the certainty of evidence is low. Evidence for PONV, ileus, lactation, and postpartum depression prevention is indirect or still under study. The next step should not be routine adoption, but rigorous multicenter trials with better reporting, formal blinding assessment, and longer postpartum follow-up.
