# Supplementary material for: Transcutaneous auricular vagus nerve stimulation after cesarean delivery: current evidence and trial design priorities
Source: Front Med (Lausanne). 2026 Sep 16;13:1910274. doi: 10.3389/fmed.2026.1910274 (PMC13624864; doi:10.3389/fmed.2026.1910274)
Supplement: Supplementary file 1 [file Datasheet1.docx]

**Supplementary File S1**

## 1. PubMed (NCBI)

(("transcutaneous auricular vagus"[tiab] OR "taVNS"[tiab] OR "tVNS"[tiab] OR "aVNS"[tiab] OR "auricular vagal"[tiab] OR "auricular branch of vagus"[tiab] OR "cymba conchae"[tiab] OR "cavum conchae"[tiab] OR "transauricular"[tiab] OR ("Vagus Nerve Stimulation"[MeSH] AND (auricular[tiab] OR transcutaneous[tiab]))))
AND
(("Cesarean Section"[MeSH] OR "Postpartum Period"[MeSH] OR "Lactation"[MeSH] OR "Breast Feeding"[MeSH] OR "Depression, Postpartum"[MeSH] OR "Anesthesia, Obstetrical"[MeSH] OR caesarean*[tiab] OR cesarean*[tiab] OR postpartum*[tiab] OR puerperi*[tiab] OR obstetric*[tiab] OR "perinatal depression"[tiab] OR lactation*[tiab] OR breastfeed*[tiab] OR "breast feeding"[tiab])
OR
("Postoperative Nausea and Vomiting"[MeSH] OR "Ileus"[MeSH] OR "Pain, Postoperative"[MeSH] OR "Analgesia, Patient-Controlled"[MeSH] OR "Oxytocin"[MeSH] OR PONV[tiab] OR "postoperative ileus"[tiab] OR afterpain*[tiab] OR "after pain"[tiab] OR "opioid sparing"[tiab] OR "opioid-sparing"[tiab] OR "Edinburgh Postnatal Depression"[tiab] OR EPDS[tiab] OR "milk ejection"[tiab] OR oxytocin[tiab]))

## 2. Embase (Elsevier)

('transcutaneous auricular vagus':ab,ti OR taVNS:ab,ti OR tVNS:ab,ti OR aVNS:ab,ti OR 'auricular vagal':ab,ti OR 'auricular branch of vagus':ab,ti OR 'cymba conchae':ab,ti OR 'cavum conchae':ab,ti OR transauricular:ab,ti OR 'vagus nerve stimulation'/exp)
AND
(('cesarean section'/exp OR 'puerperium'/exp OR 'lactation'/exp OR 'breast feeding'/exp OR 'puerperal depression'/exp OR 'obstetric anesthesia'/exp OR caesarean*:ab,ti OR cesarean*:ab,ti OR postpartum:ab,ti OR puerperi*:ab,ti OR obstetric*:ab,ti OR lactation:ab,ti OR breastfeeding:ab,ti OR 'perinatal depression':ab,ti)
OR
('postoperative nausea and vomiting'/exp OR 'paralytic ileus'/exp OR 'postoperative pain'/exp OR 'patient controlled analgesia'/exp OR 'oxytocin'/exp OR PONV:ab,ti OR 'postoperative ileus':ab,ti OR afterpain*:ab,ti OR 'opioid sparing':ab,ti OR EPDS:ab,ti OR 'milk ejection':ab,ti))

## 3. Web of Science Core Collection (Clarivate)

TS=("transcutaneous auricular vagus" OR "taVNS" OR "tVNS" OR "aVNS" OR "auricular vagal" OR "auricular branch of vagus" OR "cymba conchae" OR "cavum conchae" OR "transauricular")
AND
TS=(caesarean OR cesarean OR postpartum OR puerperi* OR obstetric* OR lactation OR breastfeed* OR "perinatal depression" OR "postoperative nausea" OR "postoperative ileus" OR "postoperative pain" OR afterpain* OR PONV OR EPDS OR "milk ejection" OR oxytocin)

## 4. Cochrane Library — CENTRAL (Wiley)

#1 ("transcutaneous auricular vagus" OR taVNS OR tVNS OR aVNS OR "auricular branch of vagus" OR "cymba conchae" OR "cavum conchae"):ti,ab,kw
#2 MeSH descriptor: [Vagus Nerve Stimulation] explode all trees
#3 #1 OR #2
#4 (caesarean OR cesarean OR postpartum OR obstetric OR lactation OR breastfeed* OR "postoperative nausea" OR "postoperative ileus" OR "postoperative pain" OR afterpain* OR oxytocin):ti,ab,kw
#5 #3 AND #4

## 5. ClinicalTrials.gov (U.S. NIH)

Other terms: "transcutaneous auricular vagus" OR taVNS OR "auricular vagus nerve stimulation"
Condition or disease: caesarean OR cesarean OR postpartum OR obstetric OR "postoperative pain" OR "postoperative nausea" OR "postoperative ileus"

## 6. WHO International Clinical Trials Registry Platform (ICTRP)

Title: taVNS OR "auricular vagus" OR "transcutaneous auricular vagus"
Condition: caesarean OR postpartum OR obstetric OR postoperative

## 7. Chinese Clinical Trial Registry (ChiCTR)

Intervention (干预措施): 经皮耳迷走神经刺激 OR taVNS OR 耳迷走神经
Condition (适应症): 剖宫产 OR 产后 OR 围术期 OR 术后疼痛 OR 术后恶心呕吐

## 8. China National Knowledge Infrastructure (CNKI)

Subject (主题): 经皮耳迷走神经刺激 OR taVNS OR 耳迷走神经 OR 耳甲
AND
Subject (主题): 剖宫产 OR 产后 OR 围术期 OR 产后抑郁 OR 术后疼痛 OR 术后恶心呕吐 OR 术后肠麻痹 OR 哺乳 OR 催产素

## 9. Wanfang Data

Title/Keyword/Abstract (题名/关键词/摘要):
(经皮耳迷走神经刺激 OR taVNS OR 耳迷走神经 OR 耳甲)
AND
(剖宫产 OR 产后 OR 围术期 OR 产后抑郁 OR 术后疼痛 OR 术后恶心呕吐 OR 术后肠麻痹)
