# Supplementary material for: Transcutaneous auricular vagus nerve stimulation after cesarean delivery: current evidence and trial design priorities
Source: Front Med (Lausanne). 2026 Sep 16;13:1910274. doi: 10.3389/fmed.2026.1910274 (PMC13624864; doi:10.3389/fmed.2026.1910274)
Supplement: Supplementary file 2 [file Datasheet2.docx]

**Supplementary File S2 — SANRA Self-Assessment**

SANRA Self-Assessment of Reporting Quality

**SANRA scoring key (per item):**

**0** = not met (item is absent or insufficiently addressed)
**1** = partially met (item is addressed but with notable limitations)
**2** = fully met (item is comprehensively and explicitly addressed)

**Maximum total score:** 12 (6 items × 2).

| **#** | **SANRA item** | **Self-score** | **Conservative justification** |  |
| --- | --- | --- | --- | --- |
| 1 | Justification of the article importance | 2 / 2 | The Introduction explains why post-caesarean pain, nausea, breastfeeding concerns and early mood symptoms are relevant to perioperative medicine and obstetric anaesthesia. The importance is framed as a research question, not as a claim of established therapy. | |
| 2 | Concrete aims or questions | 2 / 2 | The final paragraph of the Introduction states four questions: direct obstetric evidence, indirect perioperative evidence, plausible mechanisms, and trial design needs. | |
| 3 | Description of literature search | 1 / 2 | The Review lists the databases, registries, Chinese sources, search terms and absence of language/date restrictions. The revised manuscript also explains duplicate checking, screening, verification of inclusion and extraction, and resolution of differences. Exact retrieval, deduplication and exclusion counts were not prospectively recorded and are acknowledged as unavailable. | |
| 4 | Referencing | 1 / 2 | The reference list contains numbered entries with DOI and PMID information where available. The cited sources are separated by evidentiary role: background, direct obstetric evidence, indirect perioperative evidence, preclinical mechanisms, and trial-methodology literature. | |
| 5 | Scientific reasoning | 1 / 2 | The manuscript separates the single direct obstetric RCT from indirect surgical, psychiatric, healthy-volunteer and preclinical evidence. Mechanistic statements are described as hypotheses. Certainty remains limited because only one direct obstetric efficacy trial is available. | |
| 6 | Appropriate presentation of data | 1 / 2 | The main trial effect estimate is reported with a confidence interval. Data were not pooled because the included studies differed markedly in population, stimulation protocol and outcome. | |
| SANRA total | | 8 / 12 | This score reflects a structured narrative Review. It is not a systematic review or meta-analysis. The main methodological limitations are the absence of a prospectively maintained screening log, no fully independent duplicate screening, heterogeneous evidence and dependence on one direct obstetric efficacy trial. | |

**Interpretation. The score is a self-check, not a claim of high methodological quality. The main limitations are the narrative format, the absence of a prospectively maintained record log, heterogeneous evidence, and dependence on one direct obstetric efficacy trial.**
